# Nickel(II) sulphate–induced allergic contact dermatitis as experimental tool to investigate inflammatory pruritus in humans

**DOI:** 10.3389/falgy.2026.1786200

**Published:** 2026-04-22

**Authors:** Karoline Lukaschek, Kiran Kumar Bali, Konstantin Agelopoulos, Mustafa Kaplan, Sonja Ständer, Roman Rukwied, Elke Weisshaar

**Affiliations:** 1 Division of Occupational Dermatology, Department of Dermatology, Ruprecht-Karls University Heidelberg, Heidelberg, Germany; 2 Mannheim Centre for Translational Neuroscience (MCTN), Medical Faculty Mannheim, University Heidelberg, Heidelberg, Germany; 3 Section Pruritus Medicine and Centre for Chronic Pruritus, Department of Dermatology, University Hospital Münster, Münster, Germany

**Keywords:** contact allergy, itch, microdialysis, microRNAs, nerve fibre density, non-coding RNAs, translational dermatology

## Abstract

Allergic contact dermatitis is a leading cause of occupational skin disease, with nickel(II) sulphate representing one of the most prevalent contact allergies worldwide. Clinically, nickel-induced dermatitis is characterised by pronounced inflammation and intense pruritus. The functional role of endogenous mediators, structural neuronal changes, and molecular mediators contributing to the generation of itch in allergic contact dermatitis still needs to be investigated. We present nickel(II) sulphate-induced contact dermatitis as a mechanistic model to investigate pruritus under controlled conditions in humans. Thereby, we can combine and correlate clinical characterisation of nickel(II) sulphate contact dermatitis with psycho-physical, structural, and molecular analyses to identify inflammatory pathways, mediator profiles, and gene regulatory pathways involved in pruritus generation. By enabling the systematic characterisation of itch mechanisms at molecular, structural, and functional, levels, this approach provides a translational scope to advance our understanding of pruritogenic pathways and for developing targeted therapeutic strategies in allergic contact dermatitis.

## Introduction

Allergic contact dermatitis is a leading cause of occupational skin disease, with nickel(II) sulphate being one of the most prevalent and clinically relevant contact allergies worldwide (1, 2). Nickel-induced allergic contact dermatitis is characterised by severe pruritus; however, the underlying mechanistic link is not fully understood. In contact dermatitis, the mediators and neuronal processes specifically underlying pruritus have been characterised only in part (3–6). Still open questions comprise the contribution of endogenous pruritogens, alterations in cutaneous sensory innervation, and the regulatory impact of the non-coding RNAs, such as micro-ribonucleic acids (microRNAs), on neuroimmune signalling.

The functional relevance of microRNAs in regulating itch-associated mediators in allergic contact dermatitis on transcriptional level, in particular, has not yet been elucidated. Consequently, therapeutic strategies for chronic pruritus remain largely symptomatic and predominantly anti-inflammatory, without targeting the causal pathomechanisms of itch (7, 8).

Recent findings highlight a complex cutaneous neuro-immune interplay between keratinocytes, immune cells, and peripheral sensory nerve endings, contributing to the release of pruritogenic mediators and enhanced neural sensitivity in inflammatory dermatoses. Relevant signalling pathways include thymic stromal lymphopoietin (TSLP), interleukin-31 (IL-31), as well as transient receptor potential (TRP) and protease-activated receptor (PAR)-dependent mechanisms (4, 9, 10). However, experimental models in humans that would enable an integrated investigation of these pathways and regulatory mechanisms of pruritus remain scarce, and the present work aims to establish allergic contact dermatitis induced by nickel(II) sulphate as a suitable experimental human model. To this end, participants with known nickel sensitisation were experimentally re-exposed to nickel(II) sulphate to induce allergic contact dermatitis. Intraepidermal nerve fibre density was quantified in lesional and non-lesional skin, neuroperception was assessed using transcutaneous electrical stimulation paradigms, and dermal microdialysis samples were obtained from lesional and non-lesional skin to allow for interstitial microRNA expression pattern analyses.

This publication does not present a full dataset of the study results; rather, its purpose is to highlight nickel(II) sulphate-induced allergic contact dermatitis as a potentially valuable human research tool to investigate structural and gene regulatory mechanisms involved in itch—and, particularly, might be a conceptual starting point for early-career researchers in occupational dermatology.

## Methods

### Study procedures

The study was conducted in accordance with the Declaration of Helsinki and applicable national guidelines on patient safety and research ethics (11). The study protocol was approved by the Ethics Committee of the Medical Faculty of the Ruprecht-Karls University Heidelberg (S-392/2021). All participants provided written informed consent to participate after receiving detailed oral and written information about the study. Participation was voluntary, and participants could withdraw their consent at any time without providing reasons and without any negative impact on their medical care.

All clinical data, photographic documentation, and study-related observations were recorded using Research Electronic Data Capture (REDCap), a secure, web-based software platform designed for the collection, management, and processing of research data in clinical and translational studies.

Participants with a history of nickel contact allergy were recruited through the Division of Occupational Dermatology, Department of Dermatology, Ruprecht-Karls University Heidelberg, as well as via public advertisement. Interested individuals received detailed information about the study aims and procedures during an initial telephone interview. Participants received financial compensation for their time and effort. Reimbursement included travel costs for participants to the study centre at the Division of Occupational Dermatology, University of Heidelberg, and was adjusted to the volunteers' attendance, taking part in one (biopsy) or both (biopsy plus microdialysis) sessions of assessment.

### Inclusion and exclusion criteria

The inclusion criteria were as follows:

- age between 18 and 64 years
- capacity to provide informed consent
- sufficient command of the German language
- absence of active skin diseases or lesions (e.g., eczema).

Exclusion criteria comprised:

- current treatment with antipsychotics or antidepressants
- severe systemic diseases (e.g., neurological or oncological disorders)
- topical or systemic use of antihistamines, corticosteroids, immunosuppressants, or biologics

### Clinical assessments

All examinations were conducted in the Division of Occupational Dermatology. At study entry, each participant underwent a complete physical examination. Standardised and validated instruments were used to assess patient-reported outcomes. In addition to demographic and socioeconomic baseline characteristics (age, sex, marital status, employment, number of children, and highest educational level), participants were asked whether they had experienced pruritus during the previous six weeks. In cases of a positive response, the 5-D Itch Questionnaire (5-DIQ) (12) was used to evaluate itch frequency and its impact on daily activities. Further assessments included current stress level, measured using a numerical rating scale (0–10) (13), monitoring health-related quality of life, which was measured with the Short Form-12 Health Survey (SF-12) (14), and symptoms of anxiety and depression, evaluated using the Hospital Anxiety and Depression Scale (HADS) (15, 16).

### Induction of contact dermatitis

To enable structural (skin biopsy) and microRNA (intradermal microdialysis) investigations, allergic contact dermatitis was experimentally induced at two different anatomical sites, i.e., the upper back and the forearm skin.

**Figure 1:**
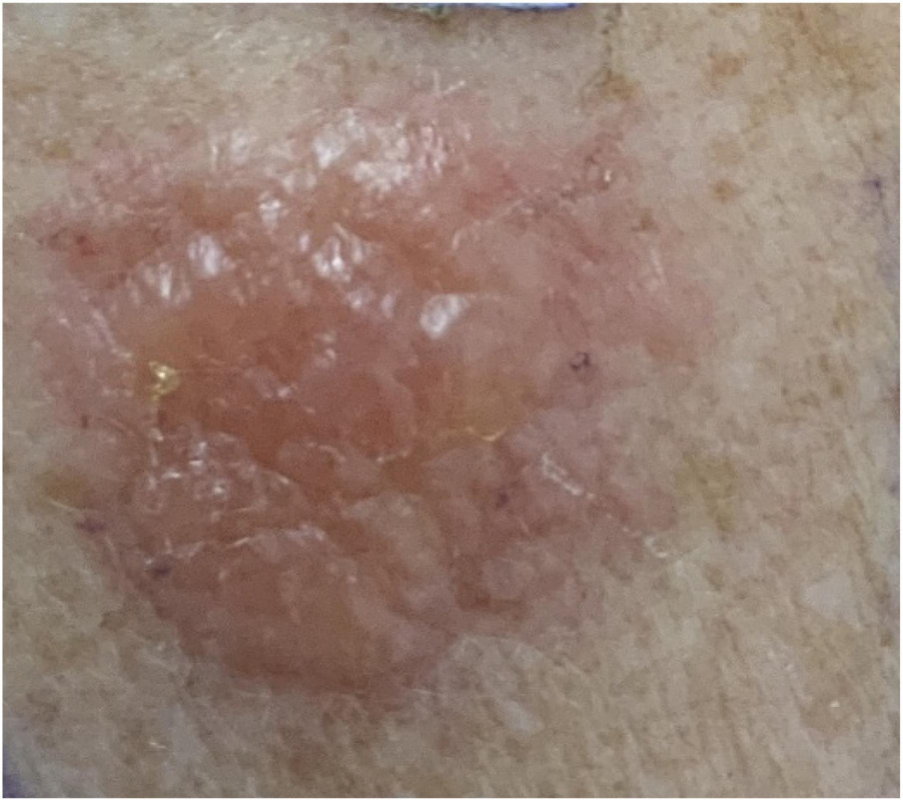
Induction of allergic contact dermatitis with a very strong (+++) patch test reaction to nickel(II) sulphate at 72 h.

For skin biopsy, a standard patch-test preparation containing 5% nickel(II) sulphate in white petrolatum (SmartPractice Europe GmbH, Greven, Germany) was applied on clean skin, using an 8 mm Finn Chamber on Scanpor (SmartPractice Europe GmbH, Greven, Germany; Figure 1). The chamber was placed on the upper back and left *in situ* for 48 h.

For the microdialysis procedure**,** allergic contact dermatitis was induced on the volar forearm using a large 18 mm Finn Chamber on Scanpor, similarly by means of 5% nickel(II) sulphate in white petrolatum applied for 48 h.

Participants were instructed to keep both application areas dry and to avoid physical activity during the exposure period. After 48 h, the patches were removed, and cutaneous reactions were evaluated at 48 and 72 h post-application.

### Skin biopsy

On the third study day (72 h reading), photographic documentation of the test site on the upper back was carried out, and a skin biopsy was obtained. A standard 3 mm punch biopsy (Stiefel Biopsy punch, Stiefel GSK) of the skin was taken under local anaesthesia with intracutaneous 1% Scandicain® following 5 min post injection. One biopsy was collected from the experimentally induced allergic contact dermatitis (“lesional”) skin site and a second from clinically unaffected skin (“non-lesional”) located approximately 3 cm distally. Wound closure was performed using one to two single interrupted sutures with 4-0 Prolene, followed by application of a pressure dressing. Suture removal was carried out by the participant's general practitioner after approximately ten days.

Tissue samples were immediately fixed in 4% paraformaldehyde (Morphisto; phosphate-buffered saline, pH 7.2) and subsequently sent to the Laboratory of the Centre for Chronic Pruritus (KCP), Department of Dermatology, University Hospital Münster, for histological and molecular-biological analyses (epidermal nerve fibre branching).

### Electrostimulation

**Figure 2:**
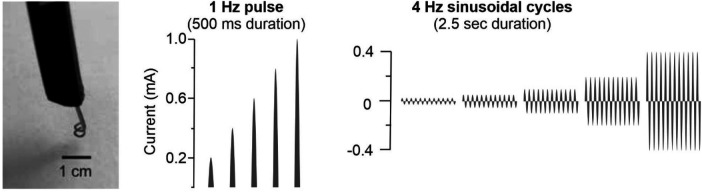
Electrical stimulation using a bipolar electrode (left). A single sinusoidal pulse of 1 Hz (middle) and 500 ms duration or continuous 4 Hz sinusoidal cycles (right) of 2.5 s duration (10 pulses in total) were delivered at random intensities of 0.2–1 mA (1 Hz) or 0.025–0.4 mA (4 Hz) for C-nociceptor activation.

To activate C-nociceptors, which play a key role in the neural processing of itch, transcutaneous electrical stimulation was performed using sinusoidal pulses at frequencies of 1 Hz and 4 Hz (Figure 2). This electrical stimulation paradigm has been shown to evoke itch in patients with atopic dermatitis (17). Electrical sinusoidal pulses were delivered transcutaneously by a pair of rounded bipolar platinum electrodes (diameter 0.4 mm, distance 2 mm) and generated by a constant current stimulator (Digitimer DS5, Welwyn Garden City, UK) connected to a Digital-Analogue Converter (DAQ NI USB-6221, National Instruments, Austin, TX, USA) controlled by Dapsys 8 software (https://www.dapsys.net).

**Figure 3:**
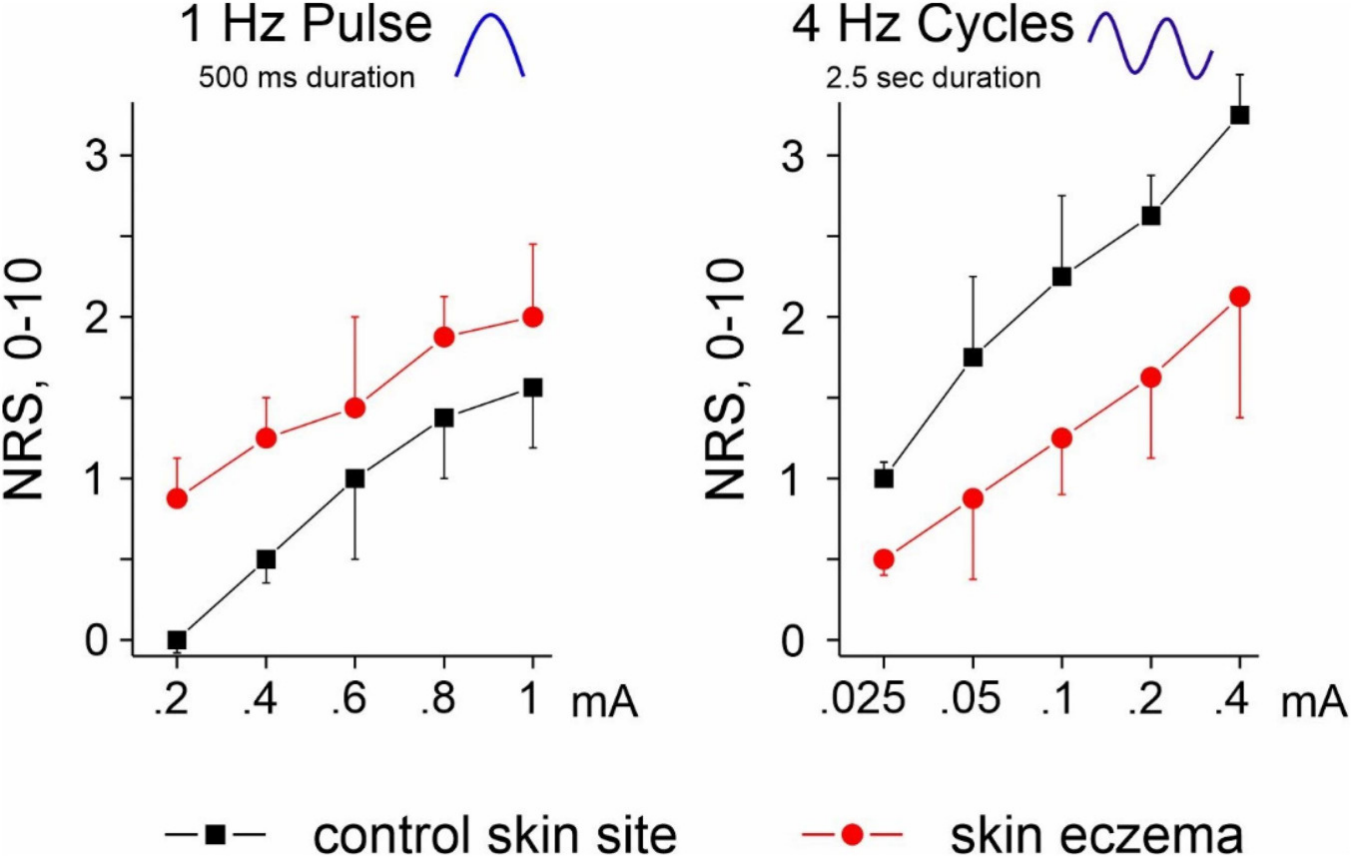
Magnitude sensation (Numeric Rating Scale, NRS) upon electrical stimulation delivering a single sinusoidal pulse of 1 Hz (500 ms, left panel) at intensities of 0.2–1 mA and 4 Hz sinusoidal cycles (2.5 s, right panel) of 0.025–0.4 mA amplitudes. Evoked sensation at control skin site: pure pain; in eczema: a mixture of pain and itching.

Transcutaneous electrical sinusoidal stimulation elicited pain in normal skin and itching in the eczema lesion in 70% of patients. The current threshold required to elicit a sensation was higher in eczema than in normal skin (0.072 ± 0.03 mA vs. 0.026 ± 0.01 mA). Maximum intensity of sensation during electrostimulation was NRS 2 in eczema and NRS 3 in control skin (Figure 3). In the eczema lesion, the sensation was a mixture of itching and pain; in control skin, pure stinging pain.

### Intradermal microdialysis

**Figure 4:**
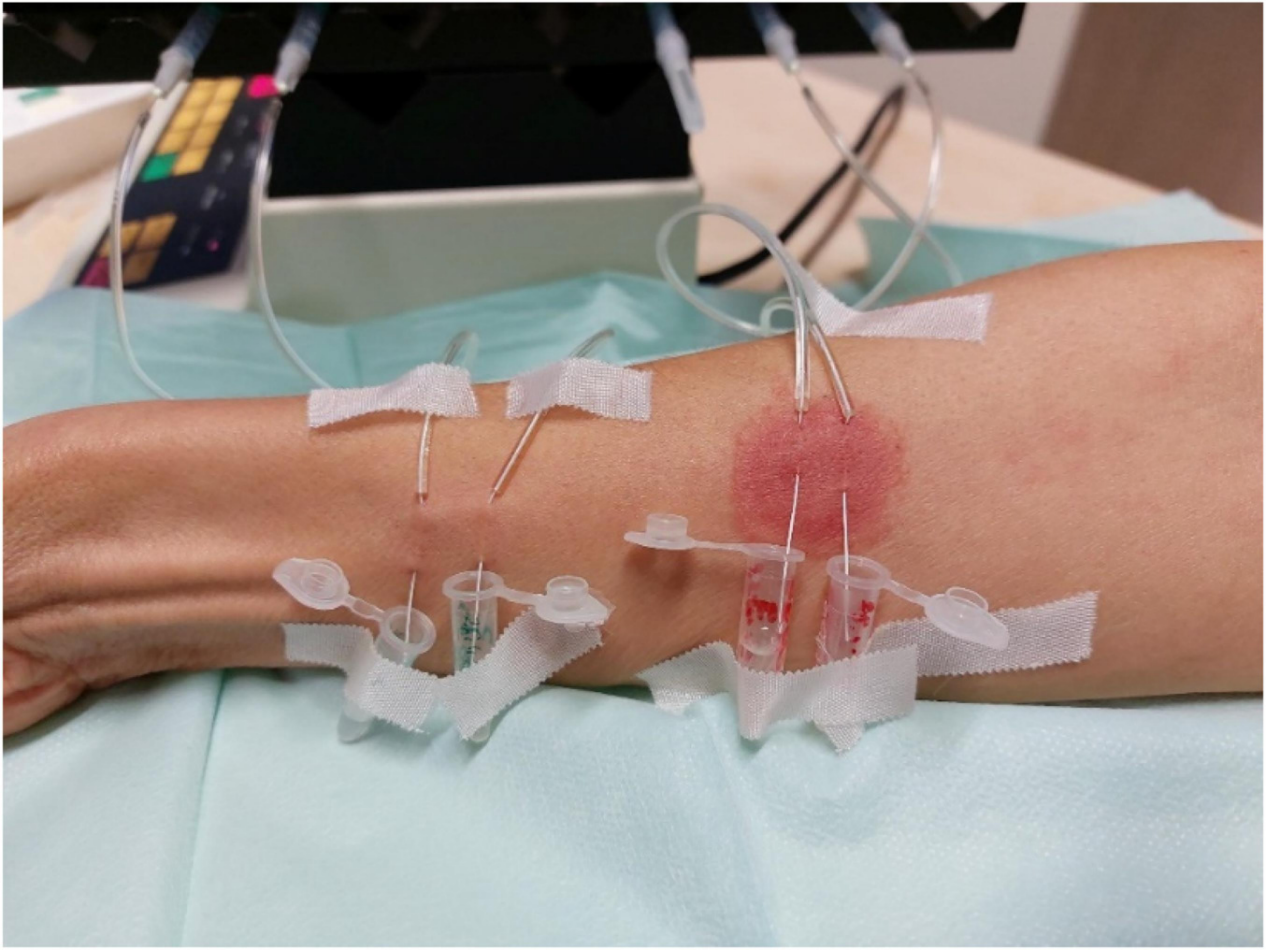
Intraepidermal microdialysis in a participant with nickel(II) sulphate–induced contact dermatitis. Microdialysis catheters were continuously perfused with 0.9% NaCl at a flow rate of 4 µL/min. After skin passage, the perfusate was collected in Eppendorf vials and, following a sample period of 60 min, samples were snap frozen in liquid nitrogen and stored at −80 °C.

To collect interstitial fluid for the analyses of extracellular mediators and microRNAs, dermal microdialysis was conducted. A microdialysis catheter equipped with a semipermeable membrane (3,000 kDa cut-off) was inserted intracutaneously at a length of 1.5 cm by means of a 25G cannula in the volar forearm. Local anaesthesia was not required. Four fibres were inserted into the skin, two placed inside the 5% nickel(II) sulphate “lesional” site and two fibres 5–10 cm distal in “non-lesional” skin (Figure 4).

The dialysis membrane allowed diffusion of small molecules between the tissue and the saline perfusate. Thereby, the relative recovery of molecules in the saline perfusate is determined by the flow rate of perfusion, the length of the microdialysis catheter in the skin, and the capacity of the molecule to bind to skin proteins. Flow rate was maintained at a constant 4 µL/min using a precision microdialysis pump (Pump 22, Harvard Apparatus, USA) to ensure stable perfusion conditions, and the catheter length in the skin was 1.5–2 cm. Fluid samples were collected after passage through the skin in RNAse-Free microcentrifuge tubes, immediately frozen in liquid nitrogen, and subsequently stored at −80 °C until microRNA analyses were performed by the Mannheim Centre for Translational Neuroscience and associated partners.

### Isolation of total RNA from skin microdialysate

**Figure 5:**
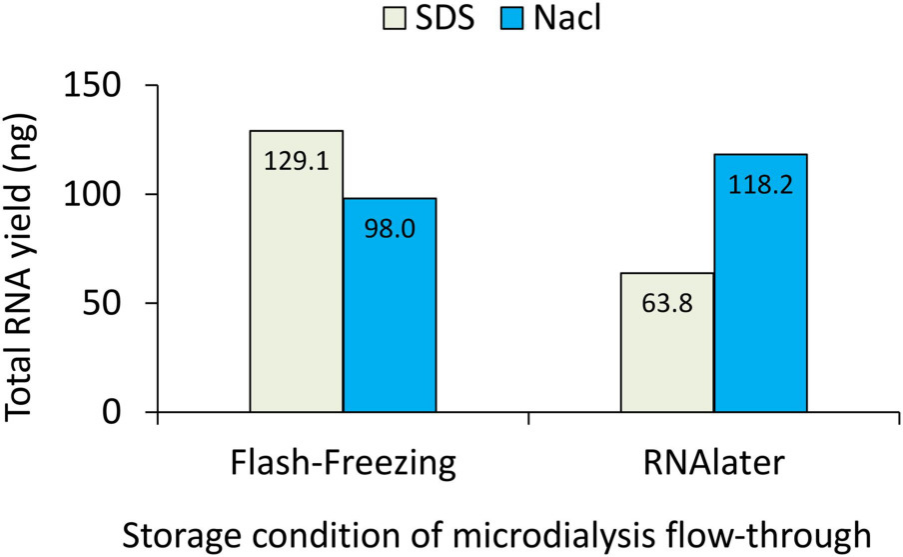
Total RNA yield from skin microdialysis flow-through under different buffer and storage conditions. The total yield (ng) was compared between microdialysate collected in 0.5% SDS-containing buffer or NaCl buffer and either flash-frozen immediately or stored in RNAlater prior to sequencing library preparation.

To establish a robust protocol for isolating total RNA from the low volume of the skin microdialysate available (∼200 μL), we compared different collection buffers and post-collection storage conditions. As shown in Figure 5, total RNA yield from microdialysis flow-through varied depending on both buffer composition and storage condition.

Under flash-freezing, samples collected in SDS-containing buffer yielded more RNA than those collected in NaCl buffer (129.1 ng vs. 98.0 ng, Figure 5). When the samples were immediately mixed with RNAlater (RNA stabilizing reagent usually used for tissue storage, Thermofisher, AM7020), RNA yield was lower in SDS samples (63.8 ng) but slightly increased in NaCl samples (118.2 ng). The higher yield observed in SDS samples may reflect cellular damage and the release of intracellular material. In contrast, the reduction in RNA yield after RNAlater storage in SDS-containing samples, together with the increase obeserved in NaCl samples, suggests that the stabilizing solution may have affected RNA recovery and its quality. To analyse non-coding RNA expression in the interstitial space, NaCl buffer and immediate flash-freezing in liquid nitrogen at the end of collection were selected. This approach minimised potential intracellular contamination and avoided changes in RNA quantity and quality associated with RNAlater.

### Determination of intraepidermal nerve fibre density

**Figure 6:**
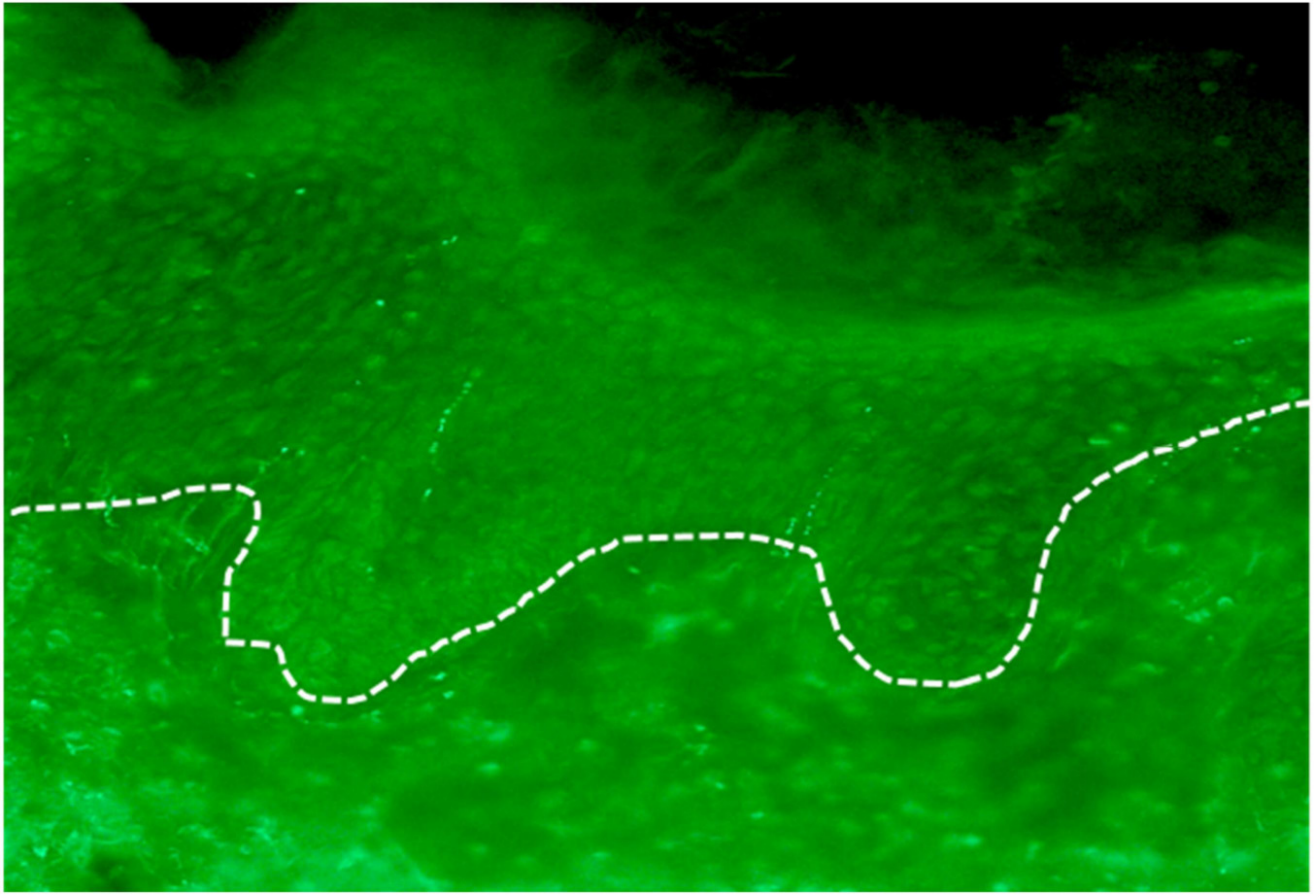
Microscopic analysis of skin biopsies for determination of intraepidermal nerve fibre density.

Quantification of intraepidermal nerve fibre density (IENFD) was performed as previously described (18). Nerve fibres were visualised in 30 µm paraformaldehyde-fixed cryosections by immunofluorescent staining for the pan-axonal marker PGP 9.5. Fluorescence imaging was carried out along the basement membrane and vertically through the full section depth to identify each nerve fibre crossing the basement membrane and its branching pattern (Figure 6). For IENFD quantification, only fibres clearly crossing the basement membrane were counted.

## Data processing and statistical analysis

Clinical and patient-reported data were recorded and managed using REDCap, ensuring standardised data capture. Psychophysical data were analysed using non-parametric Wilcoxon's matched-pairs comparisons (signed rank test). Structural and molecular data were collected using standardised laboratory protocols and procedures as described above and analysed using non-parametric Mann–Whitney *U*-tests. Notably, as this study aims to introduce the experimental model and assess feasibility, no inferential statistical analyses were performed. Continuous variables are presented as means ± standard deviation, and categorical variables as absolute numbers and percentages.

## Conclusions and perspective

A total of 20 participants were enrolled in the study (70% female; mean age ± SD: 44.4 ± 9.9 years). All patients exhibited a ++/+++ positive patch test reaction to nickel(II) sulphate. The majority showed an atopic skin diathesis (*n* = 13) and metal intolerance (*n* = 15). All patients reported pruritus (numeric rating scale ranging 2–8) at 24 h of patch application. Analyses of the microdialysis samples proved successful in (a) isolating very low quantity of RNA present in the micodialysis samples collected from the interstitial space in the skin and (b) standardizing the protocol to obtain *bonafide* sequencing libraries despite low input RNA. Ongoing microRNA sequencing will identify differentially regulated microRNAs in lesioned skin as compared to the healthy skin and potential target genes involved in regulating allergic contact dermatitis itch.

Nickel(II) sulphate–induced allergic contact dermatitis proved to be a reproducible and practicable human model for investigating pruritus, offering a valuable translational bridge between experimental research and clinical application. All individuals who had a history of contact dermatitis or a positive patch-test reaction to nickel(II) sulphate developed allergic contact dermatitis upon re-exposure during the study, and all reported intense to very intense pruritus in the affected areas. The observed alterations in cutaneous neuronal innervation patterns resemble those previously reported in patients with chronic pruritus (18). These initial findings in correlation to differentially microRNA regulated genes in “lesional” skin may provide mechanistic insights into the pronounced pruritus typically associated with nickel-induced contact dermatitis.

Electrical excitation thresholds were higher in lesional skin, possibly reflecting increased skin thickening due to contact allergy. Although the magnitude of electrically evoked sensation was lower in eczema lesions, supra-threshold stimulation induced itching in 70% of participants at this site, whereas burning pain was reported at control skin sites. Itching was observed during both 1 Hz and 4 Hz stimulation paradigms, which activate both polymodal and silent C-nociceptors (19, 20). As their differential contribution to itch cannot be resolved directly by psycho-physics, we applied a translational approach and investigated transcriptional factors in parallel in the skin during allergic contact dermatitis. This approach provides further insight into cellular regulatory processes associated with itch in contact allergy. We focused on non-coding RNAs, particularly microRNAs, in skin microdialysate due to their ability to regulate multiple pathology-relevant genes and act as master regulators (21, 22).

Despite regulatory efforts aiming at regulating the use of nickel in jewellery and other products that come into contact with the skin—such as the EU Nickel Directive (23) and its integration into the Registration, Evaluation, Authorisation and Restriction of Chemicals (REACH)-regulation—nickel contact allergy remains highly prevalent across Europe. Although the prevalence among younger women has declined following regulatory measures, nickel sensitivity continues to be one of the most common contact allergies. This persistence is primarily attributable to long-standing sensitisation in older individuals (24).

Future analyses will integrate patient-reported outcomes—assessed using the 5-D Itch Questionnaire (5-DIQ), Hospital Anxiety and Depression Scale (HADS), Short Form-12 Health Survey (SF-12), and the numerical stress rating scale—with psycho-physiological, structural and gene regulatory data derived from electrostimulation, skin biopsy and intradermal microdialysis.

By linking these complementary data domains, this approach may enable a more comprehensive characterisation of neuroimmune mechanisms underlying inflammatory itch in allergic contact dermatitis (4, 9, 10) and support integrative analyses across different biological levels. Questionnaire-based and psychophysical parameters following primary sensory afferent activation can be linked to altered skin structure in contact allergy. Changes in gene regulatory factors assessed in the interstitial fluid in this skin condition provide an additional marker of pathophysiological alterations. Assessing patients using a combination of these psychophysical, structural and transcriptional parameters represents a translational approach linking somatosensory function with transcriptional regulation and, ultimately, structural changes.

## Data Availability

All relevant data are included in the article. Further inquiries can be directed to the corresponding author.

## References

[1] Ahlström MG Thyssen JP Wennervaldt M Menné T Johansen JD. Nickel allergy and allergic contact dermatitis: a clinical review of immunology, epidemiology, exposure, and treatment. Contact Dermatitis. (2019) 81(4):227–41. 10.1111/cod.13327 31140194

[2] Schuttelaar ML Ofenloch RF Bruze M Cazzaniga S Elsner P Gonçalo M Prevalence of contact allergy to metals in the European general population with a focus on nickel and piercings: the EDEN fragrance study. Contact Dermatitis. (2018) 79(1):1–9. 10.1111/cod.12983 29635802 PMC6001707

[3] Lambert J. Itch in allergic contact dermatitis. Front Allergy. (2021) 2:702488. 10.3389/falgy.2021.702488 35386968 PMC8974693

[4] Misery L Pierre O Le Gall-Ianotto C Lebonvallet N Chernyshov PV Le Garrec R Basic mechanisms of itch. J Allergy Clin Immunol. (2023) 152(1):11–23. 10.1016/j.jaci.2023.05.004 37201903

[5] Schmelz M. How do neurons signal itch? Front Med (Lausanne). (2021) 8:643006. 10.3389/fmed.2021.643006 33791328 PMC8005641

[6] Fiebig A Leibl V Oostendorf D Lukaschek S Frömbgen J Masoudi M Peripheral signaling pathways contributing to non-histaminergic itch in humans. J Transl Med. (2023) 21(1):908. 10.1186/s12967-023-04698-z 38087354 PMC10717026

[7] Dickel H. Management of contact dermatitis. Allergo J Int. (2023) 32(3):57–76. 10.1007/s40629-023-00246-9

[8] Tramontana M Hansel K Bianchi L Sensini C Malatesta N Stingeni L. Advancing the understanding of allergic contact dermatitis: from pathophysiology to novel therapeutic approaches. Front Med (Lausanne). (2023) 10:1184289. 10.3389/fmed.2023.1184289 37283623 PMC10239928

[9] Ständer S Luger T Kim B Lerner E Metz M Adiri R Cutaneous components leading to pruritus, pain, and neurosensitivity in atopic dermatitis: a narrative review. Dermatol Ther (Heidelb). (2024) 14(1):45–57. 10.1007/s13555-023-01081-0 38182845 PMC10828226

[10] Yosipovitch G Misery L Proksch E Metz M Ständer S Schmelz M. Skin barrier damage and itch: review of mechanisms, topical management and future directions. Acta Derm-Venereol. (2019) 99(13):1201–9. 10.2340/00015555-3296 31454051

[11] The World Medical Association (WMA). WMA Declaration of Helsinki—Ethical Principles for Medical Research Involving Human Participants. France: WMA: Ferney-Voltaire (2024).

[12] Elman S Hynan L Gabriel V Mayo M. The 5-D itch scale: a new measure of pruritus. Br J Dermatol. (2010) 162(3):587–93. 10.1111/j.1365-2133.2009.09586.x 19995367 PMC2875190

[13] Karvounides D Simpson PM Davies WH Khan KA Weisman SJ Hainsworth KR. Three studies supporting the initial validation of the stress numerical rating scale-11 (stress NRS-11): a single item measure of momentary stress for adolescents and adults. Pediatr Dimens. (2016) 1(4):105–9. 10.15761/PD.1000124

[14] Ware JE Kosinski M Keller SD. A 12-item short-form health survey: construction of scales and preliminary tests of reliability and validity. Med Care. (1996) 34(3):220–33. 10.1097/00005650-199603000-00003 8628042

[15] Zigmond AS Snaith RP. The hospital anxiety and depression scale. Acta Psychiatr Scand. (1983) 67(6):361–70. 10.1111/j.1600-0447.1983.tb09716.x 6880820

[16] Snaith R Zigmond A. The hospital anxiety and depression scale. Br Med J (Clin Res ed). (1986) 292(6516):344. 10.1136/bmj.292.6516.344 3080166 PMC1339318

[17] Rukwied R Schnakenberg M Solinski HJ Schmelz M Weisshaar E. Transcutaneous slowly depolarizing currents elicit pruritus in patients with atopic dermatitis. Acta Derm-Venereol. (2020) 100(17):5906. 10.2340/00015555-3658 33026094 PMC9274932

[18] Renkhold L Wiegmann H Pfleiderer B Süer A Zeidler C Pereira MP Scratching increases epidermal neuronal branching and alters psychophysical testing responses in atopic dermatitis and brachioradial pruritus. Front Mol Neurosci. (2023) 16:1260345. 10.3389/fnmol.2023.1260345 37795274 PMC10546039

[19] Jonas R Namer B Stockinger L Chisholm K Schnakenberg M Landmann G Tuning in C-nociceptors to reveal mechanisms in chronic neuropathic pain. Ann Neurol. (2018) 83(5):945–57. 10.1002/ana.25231 29659054

[20] Rukwied R Thomas C Obreja O Werland F Kleggetveit IP Jorum E Slow depolarizing stimuli differentially activate mechanosensitive and silent C-nociceptors in human and pig skin. Pain. (2020) 161(9):2119–28. 10.1097/j.pain.0000000000001912 32379219

[21] Bali KK Kuner R. Noncoding RNAs: key molecules in understanding and treating pain. Trends Mol Med. (2014) 20(8):437–48. 10.1016/j.molmed.2014.05.006 24986063 PMC4123187

[22] Bali KK Gandla J Rangel DR Castaldi L Mouritzen P Agarwal N A genome-wide screen reveals microRNAs in peripheral sensory neurons driving painful diabetic neuropathy. Pain. (2021) 162(5):1334–51. 10.1097/j.pain.0000000000002159 33492037

[23] Garg S Thyssen J Uter W Schnuch A Johansen J Menne T Nickel allergy following European union regulation in Denmark, Germany, Italy and the UK. Br J Dermatol. (2013) 169(4):854–8. 10.1111/bjd.12556 23909687

[24] Weisshaar E Waitek M Brans R Bauer A Becker D Dickel H Assessment of the Impact of Allergies on Reduced Earning Capacity in the Context of Occupational Disease No. 5101: Nickel(II) Sulphate. Dermatol Beruf Umwelt. 2025 (Work in Progress).

